# The Effect of Processing Techniques on the Classification Accuracy of Brain-Computer Interface Systems

**DOI:** 10.3390/brainsci14121272

**Published:** 2024-12-18

**Authors:** András Adolf, Csaba Márton Köllőd, Gergely Márton, Ward Fadel, István Ulbert

**Affiliations:** 1 Roska Tamás Doctoral School of Sciences and Technology, Práter utca 50/a, 1083 Budapest, Hungary; fadel.ward@ttk.hu; 2 Faculty of Information Technology and Bionics, Pázmány Péter Catholic University, Práter utca 50/a, 1083 Budapest, Hungary; kollod.csaba@itk.ppke.hu (C.M.K.); marton.gergely@ttk.hu (G.M.); ulbert.istvan@ttk.hu (I.U.); 3 Institute of Cognitive Neuroscience and Psychology, HUN-REN Research Centre for Natural Sciences, Magyar Tudósok Körútja 2, 1117 Budapest, Hungary; 4 Department of Neurosurgery and Neurointervention, Faculty of Medicine, Semmelweis University, Amerikai út 57, 1145 Budapest, Hungary

**Keywords:** artifact rejection, brain-computer interface, electroencephalography, motor imagery, faster, CNN

## Abstract

**Background/Objectives**: Accurately classifying Electroencephalography (EEG) signals is essential for the effective operation of Brain-Computer Interfaces (BCI), which is needed for reliable neurorehabilitation applications. However, many factors in the processing pipeline can influence classification performance. The objective of this study is to assess the effects of different processing steps on classification accuracy in EEG-based BCI systems. **Methods**: This study explores the impact of various processing techniques and stages, including the FASTER algorithm for artifact rejection (AR), frequency filtering, transfer learning, and cropped training. The Physionet dataset, consisting of four motor imagery classes, was used as input due to its relatively large number of subjects. The raw EEG was tested with EEGNet and Shallow ConvNet. To examine the impact of adding a spatial dimension to the input data, we also used the Multi-branch Conv3D Net and developed two new models, Conv2D Net and Conv3D Net. **Results**: Our analysis showed that classification accuracy can be affected by many factors at every stage. Applying the AR method, for instance, can either enhance or degrade classification performance, depending on the subject and the specific network architecture. Transfer learning was effective in improving the performance of all networks for both raw and artifact-rejected data. However, the improvement in classification accuracy for artifact-rejected data was less pronounced compared to unfiltered data, resulting in reduced precision. For instance, the best classifier achieved 46.1% accuracy on unfiltered data, which increased to 63.5% with transfer learning. In the filtered case, accuracy rose from 45.5% to only 55.9% when transfer learning was applied. An unexpected outcome regarding frequency filtering was observed: networks demonstrated better classification performance when focusing on lower-frequency components. Higher frequency ranges were more discriminative for EEGNet and Shallow ConvNet, but only when cropped training was applied. **Conclusions**: The findings of this study highlight the complex interaction between processing techniques and neural network performance, emphasizing the necessity for customized processing approaches tailored to specific subjects and network architectures.

## 1. Introduction

**Figure 1:**
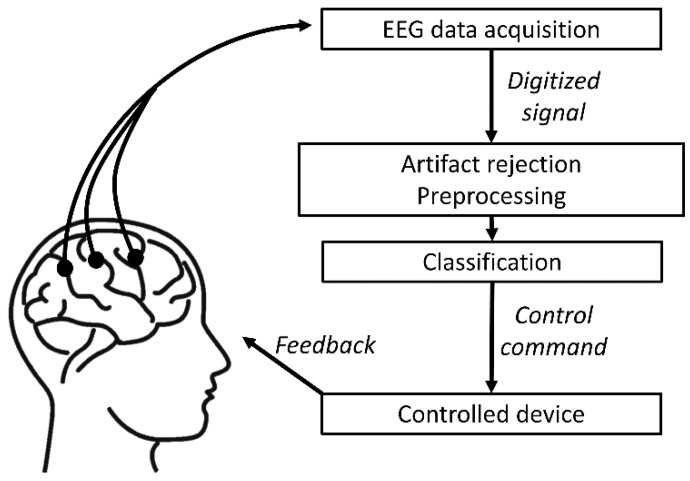
The general structure of a BCI system. Acquired data are digitized and go through preprocessing, such as artifact rejection. Afterward, a classifier decides what the intention of the user is and gives a control command based on the decision.

Brain-computer Interfaces (BCI) represent a rapidly evolving research field. As depicted in Figure 1, the general structure of these systems consists of physiological measurements of the brain, digitizing and processing the signal, and finally, giving commands based on the classification of the received data. These devices could provide significant help to people with different serious disabilities; however, reliable classification accuracy is essential for use in real life [1,2]. Non-invasive EEG measurements can be conducted more easily than invasive ones because they do not require surgery preparation. On the other hand, the classification accuracy is poorer due to the distortion from the various tissues between the source and the recording electrode and the lower resolution.

An important aspect to consider when working with EEG signals is the presence of artifacts. Artifacts refer to unwanted signals that contaminate the EEG recordings, potentially introducing significant distortions that can affect the analysis of underlying neurological phenomena [3]. It is also important to note that artifact rejection (AR) algorithms may interact with various classification methods, the interaction of which has not been well identified to date. We have limited knowledge about the extent to which artifact rejection improves (or deteriorates) the classification accuracy of various machine learning methods. Moreover, small variations in feature extraction methods may also play an important role in the classification accuracy of a BCI system. It is also unknown how small computational changes in the feature extraction methods affect the overall classification accuracy of the whole system. Finally, subjects interacting with a BCI system may not use similar strategies to achieve control over the given mental imagery task. Also, their brain anatomy and function may differ. Thus, their EEG parameters may also be different with respect to the same task; therefore, individual variations may well have a considerable effect on the classification accuracy of a BCI system. In this work, we examined how changes in the processing pipeline modify the classification accuracy of a BCI system, including individual subject variations. As the specific features that neural networks learn from are not well understood, it is possible that training the classifier interacts with the removal of artifacts and small changes in the processing pipeline. Furthermore, it cannot be guaranteed that the AR method filters out all artifactual components. It is also interesting to consider that networks trained on polluted data may also focus on the artifacts.

### 1.1. EEG Artifacts

Considering the field of EEG, two distinct categories of artifacts are recognized: physiological/biological and non-physiological. The latter can be caused by the measurement instrument, meaning faulty electrodes, powerline- and environment noise, high impedance of electrodes, cable, or body movement artifacts. These can be mostly avoided by a precise recording system and strict recording procedures [4].

Physiological artifacts may arise from various sources, including cardiac activity, pulse, respiratory patterns, and glossokinetic effects. The two most significant contributors to physiological artifacts are Electrooculograms (EOG) and Electromyograms (EMG). EOGs can come from ocular movements such as eye blinking, eye movement, and eye flatter, while EMGs can result from chewing, clenching, swallowing, sniffing, and talking [3,5].

It is important to remove artifacts from the EEG signal prior to processing, as they can potentially interfere with the interpretation of the original data.

### 1.2. Artifact Rejection for Imaginary Movement EEG Classification

To remove artifact contamination, a filtering algorithm needs to be employed. In the academic literature, several methods have been proposed for this purpose, of which the Independent Component Analysis (ICA) is one of the most frequently utilized mathematical methods [6,7,8]. In addition to ICA, other methods such as Wavelet Transformation [9], Canonical Correlation Analysis [10], Empirical-Mode Decomposition [11], and further Hybrid approaches [4] are also commonly employed. One of the widely used algorithms for AR is the FASTER algorithm [12], also utilized in this work, which, besides using the ICA method, also performs filtering and interpolation over global and epoch-vise bad channels.

### 1.3. Comparison of Artifact Rejected and Raw EEG Data Classification

To our knowledge, only a limited number of studies have examined the effects of artifact rejection (AR) methods on BCI performance, prompting our focus on the FASTER algorithm in this paper. The following studies provide relevant insights.

Zhong and Qi [13] investigated the impact of AR on P300-based BCI performance, utilizing Independent Component Analysis (ICA) for artifact removal and Bayesian Linear Discriminant Analysis (BLDA) for classification. They found AR methods can improve accuracy and information transfer rate, though their study was limited by manual filtering and a small sample of eight participants. Similarly, Kim et al. [14] assessed various AR methods (ICA, adaptive filtering, and Artifact Subspace Reconstruction) on P300/N200 classification with Support Vector Machine (SVM), finding ICA-denoised data achieved the highest accuracy at 62.87%.

Mohammadi and Mosavi [15] compared two ICA-based EOG AR methods and observed better accuracy using ICA with wavelet decomposition on the BCI Competition IV 2a dataset [16]. Winkler et al. [8] developed an ICA-based AR method for BCI use and noted minimal impact on accuracy across ERP [17] and MI-BCI [18] datasets, though individual differences were observed. Iqbal et al. [19] achieved an 80.5% accuracy using an EEGNet and Temporal Convolution Network (TCN)-based classifier with an EOG removal system, improving consistency across subjects.

Other studies explored diverse approaches to AR. Bou Assi et al. [20] combined ICA with K-means clustering for AR, observing improved accuracy from 66% to 88.1% using the Physionet database. Thompson et al. [21] found that automated AR led to a decline in P300-based BCI performance, with certain methods (SOBI, JADER, EFICA) minimizing, but not eliminating, accuracy loss. Frølich et al. [22] examined how different artifacts (e.g., blinks, muscle movements) affected motor imagery BCI and found muscle artifacts notably impacted performance, especially with broader electrode coverage.

Several studies focused on EOG-based AR. Mannan et al. [23] identified that using a lowpass filter on the EOG signal improved classification when paired with the CSP classifier on BCI Competition IV 2a data. Daly et al. [24] presented the FORCe algorithm for AR and observed it significantly improved accuracy over raw EEG, especially compared to the FASTER algorithm, in cerebral palsy patients. Merinov et al. [25] found no significant accuracy differences between four AR methods on the BCI Competition IV 2a dataset, though classification performance varied significantly between subjects.

Lastly, studies by Stigt et al. [26] and Chen et al. [27] analyzed the impact of artifact removal on EEG data. Stigt et al. observed that AR did not improve normal versus abnormal EEG classification accuracy but did slightly expedite training. Chen et al. examined specific artifact types, finding that removing EMG, powerline interference, electrode artifacts, and EOG improved accuracy by 5.5%, 4.0%, 3.1%, and 1.7%, respectively. Islam et al. [28] and Anjum et al. [29] both applied probability mapping for AR, demonstrating increased classification performance, though Anjum’s study was limited by the low electrode count of the Emotiv Pro headset used.

### 1.4. Frequency Dependence of the EEG Signal Classification

Ali Al-Saegh et al. [30] reviewed 36 studies on motor imagery EEG classification and found a strong consensus on using the 8–25 Hz frequency range associated with mu and beta bands for effective feature extraction in motor-related brain activity. Lower frequencies (0–5 Hz) were rarely used, appearing in only seven studies, indicating a preference for higher frequencies. In contrast, R. Salazar Valas and Roberto A. Vazquez [31] examined the effect of cutoff frequencies on classification accuracy with the BCI Competition IV 2a dataset and found that even the 0–10 Hz range could yield meaningful classification results, challenging the typical emphasis on higher frequencies.

### 1.5. Aim of the Study

Our article stands out by providing a comprehensive evaluation of the FASTER artifact rejection algorithm in conjunction with other preprocessing and processing methods, such as frequency filtering, transfer learning, and cropped training, across multiple neural network architectures, including EEGNet, Shallow ConvNet, and our custom classifiers. Unlike prior studies, which typically focus on a single preprocessing technique or classifier, we examine the combined effects of processing steps on classification accuracy. This approach highlights the complex interactions between artifact rejection, filtering, and neural network performance, offering unique insights into how these factors collectively influence BCI performance across different subjects and network configurations.

## 2. Materials and Methods

### 2.1. The Physionet Database-EEG Motor Movement/Imagery Dataset

We performed our research on the EEG motor movement/imagery dataset recorded by Schalk et al. as part of the Physionet Database [32]. Data were recorded with a 64-channeled 10–10 EEG system with 160 Hz sampling frequency and by using the BCI2000 framework without hardware filters [33]. It is one of the largest EEG datasets of motor imaginary tasks, consisting of recordings from 109 subjects, 14 files for each. In our work, we excluded subjects 88, 92, and 100 due to the sampling frequency and data structure mismatch. We also omitted subject 89, where labels were found to be incorrect. These problems were also reported previously [34,35].

The dataset contains five classes of motor imagery tasks, namely baseline activity and imagined activities of right-hand, left-hand, both-hand, and both-leg movements. Although the database includes EEG signals of tasks where movements were actually realized, we only used data from imagined movements to train and test the systems, as tetraplegic people are the target patients for BCI research for whom only imagined movements are available. During experiments, we used four-way classification for the following classes: right-hand, left-hand, both hands, and both legs. There are 90 motor imagery trials for each subject.

### 2.2. The FASTER Algorithm

The Fully Automated Statistical Thresholding for the EEG artifact Rejection method (FASTER algorithm) was designed by H. Nolan et al. [12]. In this chapter, the steps of this method are explained, with particular attention paid to the parts we have modified to the original algorithm.

We employed a method comprising four sequential steps, with a deviation from the original algorithm, by excluding the final artifact detection step across subjects. We chose to omit this step to avoid excluding any subjects based on artifact contamination. The algorithm utilized statistical criteria to identify channels and components that exhibited deviations exceeding three times the standard deviation of the computed parameters.

In preparation for algorithm application, frequency filtering was implemented, employing a 5th-order Butterworth filter. While the original article specified a frequency range from 1 to 95 Hz with a notch filter at 50 Hz, our study deviated by utilizing distinct frequency ranges tailored to our specific experimental parameters and objectives. The exact frequencies are detailed in Section 2.5.

The initial step of the algorithm involved the identification of globally artefactual channels. Channels exceeding predetermined thresholds for variance, the mean of the channel’s correlation coefficients with other channels, or Hurst exponent parameters were flagged as faulty. Subsequently, the algorithm proceeded to eliminate epochs containing artifacts. The examined parameters for this step included amplitude range in epoch, deviation from each channel’s average value, and variance in each epoch. The third step involved the utilization of ICA to segregate time-dependent data into statistically independent waveforms. During this process, epochs and channels labeled as defective were disregarded. A transformation was performed by using the fast-ICA algorithm. Components displaying excessive correlation with the signal of electrodes proximal to the ocular region were omitted in the resultant space, as well as components that failed to meet the Z-score criterion for kurtosis, power gradient, Hurst exponent, and median gradient parameters. In the last step, defective channels were determined on an epoch-by-epoch basis, while subsequently, both globally and individually impaired channels were replaced through the spherical spline interpolation technique. Ultimately, the data were referenced to the average of all scalp electrodes.

### 2.3. Feature Extraction and Classification

We tested different models to assess the classification accuracy. The time domain EEG was tested using EEGNet and Shallow Net. To check the effect of adding the spatial dimensions, we used the Multi-branch Conv3D approach and two newly developed models based on our dense representation of input data.

#### 2.3.1. 3D Representation of EEG Signals

**Figure 2:**
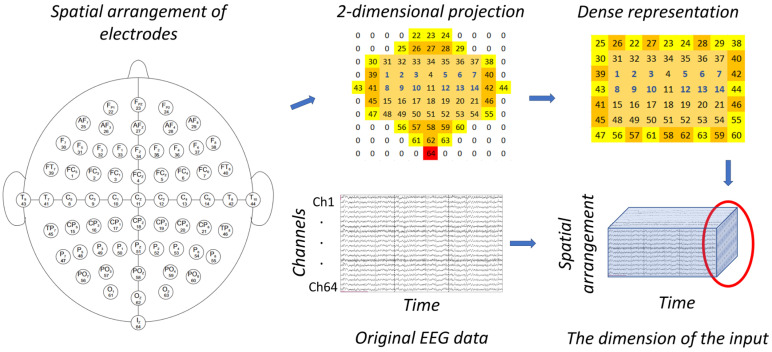
3D representation of the EEG data: the channels × time initial arrangement of the EEG data is rearranged into a three-dimensional form, where the first two dimensions refer to the spatial arrangement of the electrodes, while the third dimension is time. For the spatial dimension, we used a dense electrode arrangement. While the original distribution contains placeholder zero channels, we rearranged this map to have a dense representation with no such cells. We designed this input representation to investigate the effect of explicit involvement of the spatial arrangement of electrodes on the classification performance.

Given that the relative positions of the electrodes can carry relevant information for the classifier, we used a three-dimensional representation (two spatial dimensions and one temporal dimension) of the EEG signals as the input tensor, similar to previous studies [36,37,38]. Our goal was to examine the impact of three-dimensional representation on classification performance. For this transformation, we used a unique arrangement of electrode placements, referred to as dense 3D transformation. In this construction, the Iz electrode is omitted, and the remaining 63 electrodes are rearranged into a 9 × 7-dimensional rectangle. The arrangement can be seen in Figure 2. This arrangement will be the input shape for 2D and 3D convolutional neural networks.

#### 2.3.2. 3D CNN (Conv2D)

**Figure 3:**
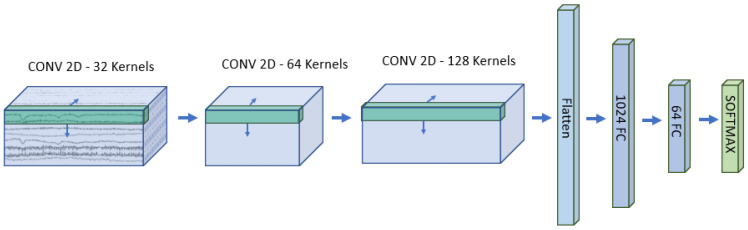
The structure of the designed 2D convolutional neural network: The input is the 3D representation of the EEG signal, on what 2D convolution is performed with 32 kernels of the size of [3 × 3 × number of timepoints] in the first layer. In the next two layers of convolution, firstly 64, then 128 kernels of dimension [3 × 3 × number of kernels of the previous layer] are performed. Next, the flattened representation is given to two layers of a fully connected network with 1024 and 64 neurons, and finally, a softmax layer is responsible for classification with the output of four numbers as the number of classes.

The first network we developed to explore the effect of 3D representation was built of 2D-convolutional layers over the original 3D data. In this operation, kernels have a dimension of 3, where the size of the 3rd dimension is equal to the number of timesteps on the time/feature dimension. With this method, the system performs convolution on the spatial arrangement, whereas, on the last axis, all data points are summed with certain weights. As the first step, L2 normalization is performed, followed by three layers of two-dimensional convolution. After the last step, the flattened data are given as input to two layers of a fully connected network, and finally, a softmax layer is responsible for the classification. The architecture can be observed in Figure 3.

#### 2.3.3. 3D CNN (Conv3D)

**Figure 4:**
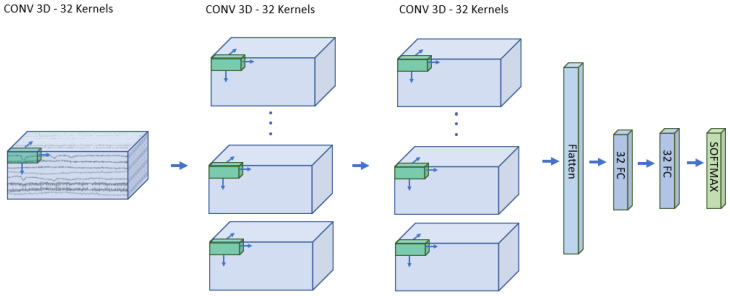
The structure of the 3D convolutional network. The input is identical to that of the 2D CNN. The difference is that in this structure, three-dimensional convolution is performed. There are 32 kernels in all the layers. The shape is (1 × 1 × 30) in the first and (2 × 2 × 40) in the second and third layers. The fully connected part consists of two layers with 32 neurons each, and finally, a softmax classification layer.

Networks with 3D convolution are widely used for video-processing tasks, and numerous articles propose this architecture for EEG classification as well [38,39]. The difference from the previous structure is that, in this case, convolution is performed even on the third dimension, resulting in a four-dimensional structure on the second layer. This requires greater memory usage but has the advantage of exploiting features scattered in time. The designed structure starts with L2 normalization. Afterward, three layers of 3D convolution are performed, with kernel dimensions [1,30] of the first layer and [2,40] for the upper layers. Between convolutions, a Batch normalization and an ELU operation are performed. As in the previous case, two layers of fully connected layers and a softmax layer are responsible for classifying the input EEG signals. Figure 4 contains the structure of this classifier.

#### 2.3.4. Multi-Branch 3D CNN

The second type of implemented 3D CNN is based on the article of Xinqiao Zhao et al. [36]. It consists of three branches, all of them with two layers of different dimensions of convolutional kernels and three layers of the fully connected network. The last layers are the size of the number of classes; thereafter, the outputs of all three branches are added up, and a softmax operation is performed for the final classification.

#### 2.3.5. EEGNet

The fourth network we used in our study is one of the most well-known classification systems for MI-signal classification, the EEGNet, presented by V. J. Lawhern et al. [40]. We used half of the sampling frequency as the length of the kernel of the first layer without applying any further modifications. The main layers of the network are a 2D convolutional layer, a depth-wise 2D convolutional layer, a separable 2D convolutional layer, and finally, a fully connected classification layer.

#### 2.3.6. Shallow Convolutional Neural Network

Finally, we evaluated the performance of the Shallow Convolutional Neural Network (Shallow ConvNet) architecture proposed by Schirmeister et al. [41]. This widely recognized algorithm has been extensively utilized in numerous studies due to its effectiveness [42,43]. This network consists of a temporal convolution, a spatial convolution, a mean pooling layer, and a linear classification FC part. The network was implemented using the source code provided in [40]. However, our implementation employs several modified parameters relative to the originally published article.

### 2.4. Transfer Learning and Fine-Tuning

A deep learning system usually requires a great amount of data to generalize features well and to have reasonable accuracy. Systems for EEG classification only acquire a limited number of samples, as recording and labeling these signals is cumbersome, requires a significant amount of time to process, and requires human intervention. The main idea of transfer learning (TL) application is pre-training a system—or a part of the system—over an independent dataset and transferring these weights as an initial state. These weights are fine-tuned during the actual training phase of the network. In the case of EEG studies, two main types of transfer learning are used [44]. One of the TL approaches is when the feature space generated from the EEG data is similar to one of those tasks for which a much larger dataset is available. An example is ImageNet, which can serve as an initial system for classifying EEG samples transformed into images [45]. The second approach, which we also used in this study, is pre-training the network on subjects that differ from the actual subject we test on. In other words, we fine-tune the globally learned network with the data from certain subjects. Multiple studies used this technique, and it yielded significant improvements in classification accuracy [2,42,44].

**Figure 5:**
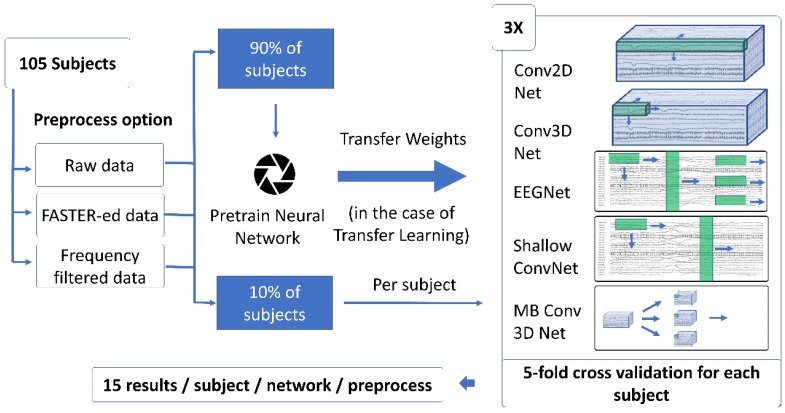
Transfer learning and the generation of results. To have statistically significant results, we performed a 5-fold cross-validation for each subject three times, resulting in 15 results for each subject, network, and preprocess option. 90% of the subjects’ data is used for pre-training the classifier, which is fine-tuned over individual subjects’ training sets. The whole process iterates 10 times for every subject to be tested.

In our study, we divided the 105 subjects of the Physionet database into two parts: a Neural Network was pre-trained over 90% of subjects, and to address inter-subject variability, the classifier was fine-tuned in the remaining subjects individually. There are 105 subjects, and we did not repeat the process 105 times, but rather 10 times, with 10% of the subjects belonging to the test set in each iteration. During pre-training, we allocated 20% of the training data as a validation set and implemented early stopping with a patience value of 15. For fine-tuning, we trained the networks for 15 epochs without using a validation dataset, as the fine-tuning dataset contained relatively few data points, making it challenging to achieve reliable validation results. The exact method for training can be observed in Figure 5.

### 2.5. Effect of Frequency Filtering

In the final phase of our study, we conducted experiments to find out the dependence of accuracy on the lowest frequency ranges (0.1 to 5 Hz) relative to the broad range of 5 to 75 Hz. We also examined the whole range of 0.1 to 75 Hz frequency band and the differences when no filtering was performed at all. To carry out this examination, we employed 5th-order band-pass Butterworth filters. The Butterworth filter, renowned for its characteristic of being maximally flat, ensures a uniform magnitude response within the pass band. However, a noteworthy drawback of this filter is the substantial width of its transition band.

### 2.6. Cropped Training

To investigate the generalizability of EEG signals with different onsets, we implemented cropped training, a method also employed by Schirrmeister et al. [41]. In this approach, the training and testing data are augmented by generating new samples through systematic time shifts of the original data. This ensures that neural networks are exposed to a broader range of temporal variations within the EEG signals, helping to simulate different onset timings and improve model generalization. In this scenario, we used 1 s long windows instead of the previous 2 s ones and performed shifts with 0.1 s long steps until 2 s. This gives 11 overlapping crops of the same epoch. Samples from a single epoch go solely to the train or solely to the test set. We repeated the previously described experiment regarding frequency dependence in this augmented dataset with all the networks, using or neglecting the FASTER method.

## 3. Results

### 3.1. The Effect of Artifact Rejection

**Figure 6:**
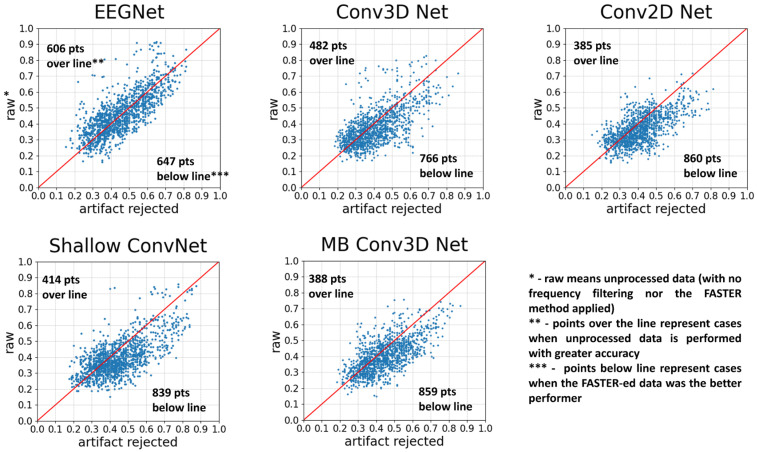
The comparison of neural networks with and without using the FASTER method. Each point represents two accuracies for a subject: the one obtained with AR and the other obtained without using the FASTER algorithm. The test was run 12 times to obtain significant results. The red line indicates the points with no difference between the two options, points over the line run with the raw option as the more accurate, and points below the line where the artifact-rejected version yields better results.

As can be observed in Figure 6 and Table 1, without using the TL method, the average of the classification accuracy of Conv 2D, Conv 3D, Shallow ConvNet, and Multi-branch Conv3D Net models are significantly improved due to the FASTER algorithm while regarding the EEGNet there was no significant improvement. In this scenario, we used 2 s long windows, and during the FASTER algorithm, a frequency filter between 0.1 and 75 Hz was applied. For the raw data, we did not use any frequency filtering.

Upon subject-specific scrutiny, it becomes evident that the influence of artifact rejection is contingent on the particular subject under evaluation. Our analysis encompassed a comprehensive approach. Initially, we executed 5-fold cross-validation three times for all four networks, both with and without artifact rejection. To substantiate disparities, we scrutinized the distribution of 15 results obtained for an individual subject with a specific network. If the data followed a normal distribution, we executed a *t*-test; conversely, if non-normality was detected, a Man–Whitney U test was performed to ascertain the significance of the observed differences.

We categorized subjects based on the extent of change in the corresponding classification performance across the various networks. Intriguingly, several scenarios arose in which certain networks led to a notable enhancement, while others yielded a significant decline in the performance for the same subjects. In response to a slight variance in results observed during a second examination, we iteratively conducted the calculations two times more to explore the evolving significance of the observed differences. Finally, we had 4 times (3 accuracy results for each cross-fold iteration and network), meaning four results of significance for each classifier.

**Figure 7:**
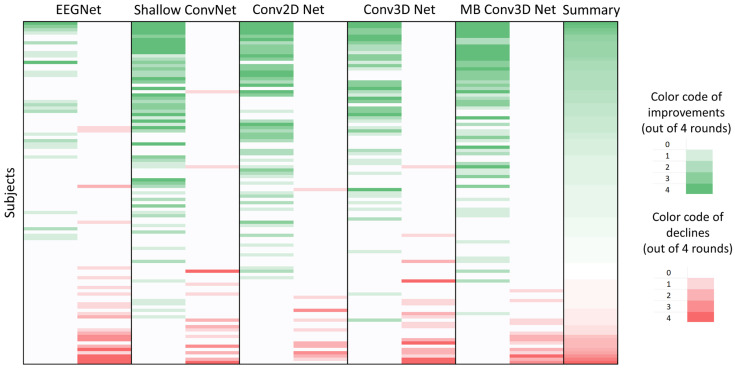
Subject dependence of artifact filtering. The shade of color yields on how many runs there were statistically significant improvement (green) or significant decline (red). (The deeper the shade, the more runs were significant.) Significance is based on the *t*-test in the case of normality and the Man–Whitney U test in the case of non-normality. Subjects are ordered by summary number, computed as the sum of the significance of improvements minus the sum of the significance of decline.

In our assessment, to each subject, a numerical score was assigned, denoted as follows: a value of −1 indicated a significant decline, 0 denoted no significant difference, and +1 represented a discernible increase observed for each computational aspect across all networks. Therefore, the cumulative score per subject ranged from −20 to 20. Twenty-five subjects scored over 8, indicating substantial performance gains from the AR method, with Subject 69 achieving a remarkable 20-point increase. Conversely, some subjects, like Subject 15, experienced significant declines, with only five subjects scoring below −8. Regarding classifiers, EEGNet showed the least improvement, with only three subjects scoring at least 3, while six scored below −3. In contrast, the other four networks had at least 20 subjects exceeding the three-point mark, with fewer than five scoring −3 or worse. The subject dependence on AR is illustrated in Figure 7.

### 3.2. The Effect of Transfer Learning

**Figure 8:**
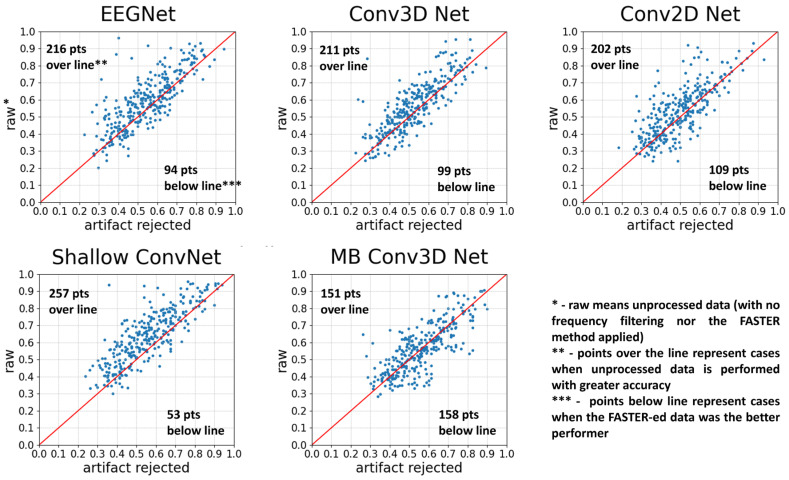
The comparison of neural networks with and without using the FASTER method, using transfer learning. Each point represents two accuracies for a subject: the one obtained with AR and the other obtained without using the FASTER algorithm. The test was run three times. The red line indicates the points with no difference between the two options, points over the line run with the raw option as the more accurate, and points below the line where the artifact-rejected version yields better results. Generally, the usage of transfer learning accuracies without artifact rejection tends to be higher.

The classification accuracy obtained by transfer learning was significantly better in every scenario. Based on the signed-rank Wilcoxon test, the learning process performs significantly better in the case of each network, both in the case of unfiltered data (Table 2) and artifact-rejected data (Table 3). In Figure 8, it can be observed that transfer learning does not improve as much in cases of artifact-rejected data as in the case of raw data, resulting in higher classification accuracies in the latter case. (This difference is significant in all the cases except the Multi-branch Conv3D Network.)

### 3.3. Comparison of Neural Networks

**Figure 9:**
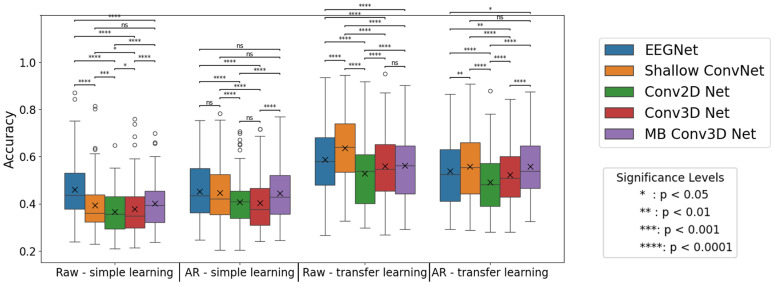
The accuracies obtained by neural networks, with and without transfer learning and artifact-rejection. In the box plot, x denotes the mean, while the horizontal line marks the median value. The limits of the box indicate the range of the central 50% of the data. Significance levels were determined using the Wilcoxon test. (The notation ‘ns’ denotes non-significant differences.) Before applying the TL method, EEG Net was the greatest performer, while after the application, Shallow ConvNet had the highest accuracy. These observations are generally true for the raw and the artifact-rejected data as well. However, for AR-ed data without TL, this difference is non-significant. The figure also presents that without transfer learning, networks generally perform better with the FASTER method, while after the application, raw data will attain a higher precision.

In network comparisons, the primary evaluative criterion centers on classification accuracy. As illustrated in Figure 9, when transfer learning is not employed, the highest classification accuracy is achieved by the EEGNet classifier, both in raw and artifact-rejected conditions. However, in the latter case, the differences between the EEGNet, Shallow ConvNet, and MB Conv3D Net were not significant. In the raw data scenario, the Multi-branch Conv3D Net is the second-best performer, followed by the Shallow ConvNet, with our proposed networks trailing.

However, the performance landscape shifts with the introduction of TL. Shallow ConvNet emerges as the top performer, surpassing EEGNet and other classifiers. In the raw data scenario with TL, EEGNet ranks second, followed by the 3D convolutional networks. In the artifact-rejected condition with TL, the MB Conv 3D CNN outperforms EEGNet, securing the shared first position, with non-significant differences from the Shallow ConvNet. This demonstrates that TL significantly influences the performance hierarchy among different network architectures.

### 3.4. The Effect of Input Representation

Using the dense 3D representation for input did not yield significant improvements in classification performance. This indicates that incorporating spatial information did not lead to higher accuracies compared to networks without this additional spatial data. The only exception is with the artifact-rejected data using transfer learning, where the MB Conv 3D CNN slightly outperformed the EEGNet.

### 3.5. The Effect of Frequency Filtering

The outcomes stemming from the frequency filtering analysis have yielded unexpected findings. We compared results using the 0.1 to 5 Hz range and the 5 to 75 Hz range. As observed in Table 4, three out of the five neural networks yielded significant results only within the first frequency range (0.1–5 Hz). Shallow ConvNet and EEGNet are the only networks for which the 5 to 75 Hz range also provides results greater than the chance level. However, for EEGNet, these results were still far below the accuracy achieved in the lower frequency range.

### 3.6. The Effect of Simple and Cropped Training

**Figure 10:**
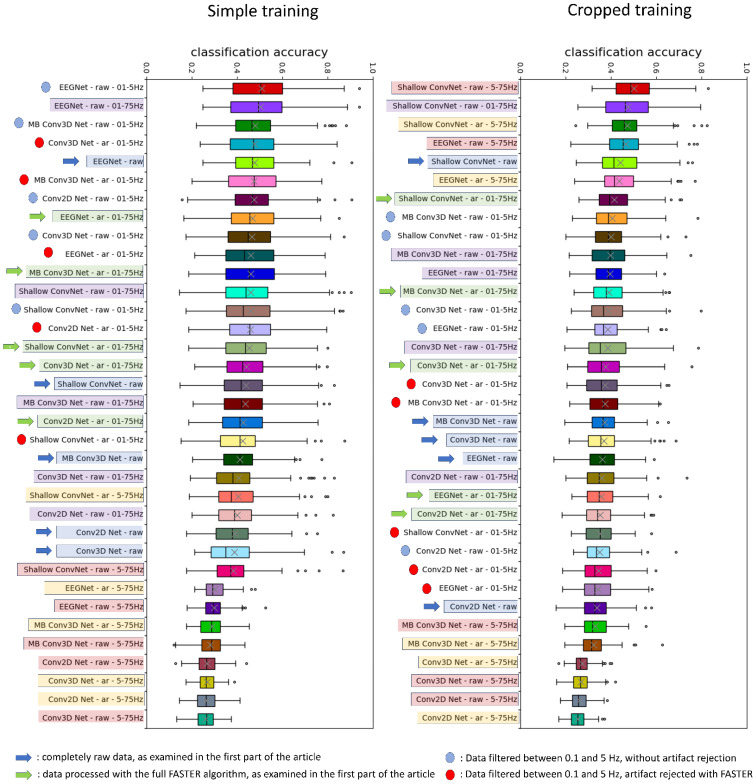
The results obtained with and without cropped training ordered by the classification accuracy. Arrows mark the cases examined in the first part of the article: the differences between the FASTER-ed (green arrow) and the fully raw data (blue arrow). The relation between the two datasets remains the same, as only the EEGNet yields a better raw classification accuracy. However, during cropped training, Shallow ConvNet also joined this group. Dots marked the results when frequency filtering was presented between 0.1 and 5 Hz. (Red dots for artifact rejected and blue dots for raw data). Without cropped training, these results tend to be unexpectedly high, yielding that this low-frequency range contains the most relevant information for MI classification on the Physionet database when subject-vise learning is performed without any augmentation of the training dataset. In the other cases, the color of the bars represents the corresponding preprocessing option. The most noteworthy result is that while during simple learning, almost every 5–75 Hz filtered attempt was presented on the lowest part of the list, the cases of EEGNet and Shallow ConvNet jumped to the first places of the cropped training results, corresponding to the extensive literature.

The results derived from simple and cropped learning can be observed in Figure 10. Upon comparing results from the simple learning process, we can state that those experiments, when 5 Hz to 75 Hz frequency filtering was performed, had the lowest accuracies. Only the Shallow ConvNet attained moderately higher accuracy, as we have observed in the previous paragraph. The relation between AR data (with 0.1 to 75 Hz frequency filter) and raw data also remains the same, as we have seen in the first part of the results: classification accuracy is enhanced by the application of artifact rejection in the case of four out of five networks, with EEGNet as the only exception. When we apply the mentioned frequency filter without the FASTER algorithm, the case of Shallow ConvNet becomes similar to the EEGNet, in the sense of being the non-artifact-rejected option, the better performer. The partly unexpected finding is the superior performance of cases where frequency filtering between 0.1 and 5 Hz was applied. These signals include only the lowest part of the frequency spectrum, meaning that they do not contain the mu or beta band.

When applying cropped training, we acquired noteworthy results, depending on the network we used for classification. Contrastingly to the simple learning approach, in the specific context of the Shallow ConvNet and the EEGNet, an inverse trend was observed where the highest performances were achieved in the broader frequency range spanning from 5 to 75 Hz. On the other hand, the worst results were attained by the higher frequency range for the other three networks, where the input feature was the 3D representation. These results show that the frequency range of the mu, beta, and gamma bands are the most valuable in the case of EEGNet and Shallow ConvNet, while the other three networks are not able to extract those features effectively. It is important to emphasize that, for the two widely used networks to achieve better results at higher frequencies, as reported in the literature, cropped training was necessary.

Within the realm of neural networks, the integration of cropped training demonstrates a parallel effect akin to the observed outcomes with transfer learning. In the absence of cropped training, EEGNet emerges as the preeminent performer. However, upon application, there is a discernible transition where Shallow ConvNet surpasses EEGNet in terms of classification prowess.

### 3.7. Subject Dependence in Simple Learning and Cropped Training

We extended our investigation to explore subject dependence within the context of cropped training. This involved conducting a series of experiments, comprising four sets of three trials each, on both the EEGNet and Shallow ConvNet systems. We selected these two networks because only these two classifiers achieved reasonable accuracy on the frequency-filtered data during cropped training. We meticulously assessed subject-wise performance, focusing on the frequency range of 5 Hz to 45 Hz for the FASTER method. As there were 4–4 significance values for both networks, each subject was assigned a point ranging from −8 to 8, facilitating a comparative analysis between FASTER-processed and completely raw data.

**Figure 11:**
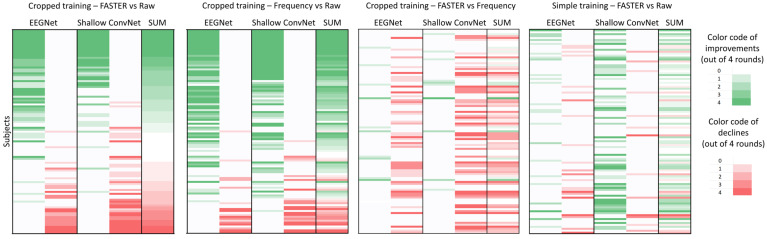
Subject-wise effect of the FASTER artifact rejection method and the frequency filtering on the classification performance of the EEGNet and Shallow ConvNet, related to the raw data, during cropped and simple training. The shade of the color means the number of significant increases (green) or declines (red) out of the four tests. Subjects are ordered by the score achieved during the AR method compared to the unfiltered data. The order of the subjects is almost the same in the second graph, meaning that frequency filtering has a similar subject-wise effect as the FASTER algorithm related to the raw data. However, this sequence is merely different from the result from the first part of the article, meaning that during cropped training, it changes on which subjects the AR has positive effects. The sequence is also different on the FASTER compared to the plain frequency filtering graph, where, for most of the subjects, the AR method caused a significant decline. It means that during cropped training, it is more advised to filter only the appropriate frequency ranges and not to use the more complex FASTER method to obtain better results.

Furthermore, alongside these comparisons, we computed accuracies based on frequency-filtered raw data spanning the 5 Hz to 45 Hz range and juxtaposed these against both raw and FASTER-processed data. These findings are synthesized and presented in Figure 11, providing valuable insights into the relative efficacy of different preprocessing techniques across various subjects. As can be observed, the FASTER-applied and frequency-filtered data exhibit similar subject-wise distributions when compared to the raw data, indicating that both methods have comparable effects on subject dependence. However, when comparing these two methods, it is evident that frequency filtering alone yields significantly higher accuracy results. Another important factor to note is that the set of subjects whose classification accuracies improved during cropped training differs substantially from those who showed improvements in the simple training process.

## 4. Discussion

As can be seen from the results, classification accuracy depends on many factors. The subject dependence of the efficacy of artifact rejection is a noteworthy result. The question that emerges is, what is the reason behind these differences? As each subject has a different scale of artifact contamination, the reasoning can be the amount and quality of contained artifacts. However, it is only partially true because, for instance, subject 15 has the greatest accuracy decline after artifact rejection, but if we examine it from closer, multiple artifactual components were rejected there. The real reason can be the number of rejected components on which neural networks can base their classification. If the data from a certain subject contain artifactual yet useful components, the accuracy will decline as a result of the application of the FASTER method.

The application of transfer learning yields a noteworthy improvement for each classification task. This beneficial effect of the process is well-documented in the existing literature [45,46,47]. What is interesting to note is that transfer learning improves the raw data classification to the greatest extent, and results obtained this way surpass the accuracy achieved on the artifact-rejected data. A plausible underlying explanation for this phenomenon may be that, with a substantial amount of training data, neural networks can learn to disregard artifacts and focus on neuronal processes. Conversely, the FASTER artifact rejection method may filter out components that are relevant and could have been utilized by neural networks for learning.

The best accuracy is achieved by the Shallow ConvNet with raw data as input and transfer learning, replacing the EEGNet, which was the classifier with the highest accuracy when transfer learning was not applied. This confirms Shallow ConvNet’s superior performance in transferring weights from the broader dataset, as was also described earlier [46].

As it was presented, adding the spatial dimension using dense 3D representation did not lead to improvements in most cases. This is likely because networks like Shallow ConvNet and EEGNet can inherently learn the spatial relationships between channels through their architectures. Shallow ConvNet achieves this via spatial filtering, while EEGNet uses DepthwiseConv2D layers. These methods allow the networks to autonomously identify and leverage the importance of spatial data and channel interactions, which proves to be more effective for classification performance than explicitly incorporating a dense 3D spatial representation.

Discussing results from the frequency filter section, the conclusion that can be drawn is when a limited number of data is given to training the classifier, such as in the case of subject-wise training, even the well-known and widely used Shallow ConvNet and EEGNet tend to concentrate on the lowest frequency ranges, which ranges can contain relevant information. This attention to lower ranges of frequency can be seen in Figure 10. An unexpected observation is that networks trained on the 0.1–5 Hz filtered dataset exhibited relatively high accuracies, often surpassing those trained on the 0.1–75 Hz filtered version. In the scenario of lowpass filtering, raw data yielded greater accuracies than the artifact-rejected data, indicating that the previously examined effect of AR does not hold under these circumstances. One possible explanation for the superior classification accuracy of the delta band is its inherently higher amplitude power compared to other frequency bands. This elevated power may provide the classifier with more prominent features, thereby enhancing performance. Additionally, the delta band is closely associated with attentional processes, which may contribute to the presence of underlying features that strongly influence classification accuracy. Furthermore, the limitations of training data without the use of cropped training or transfer learning might impair the networks’ ability to effectively recognize the importance of higher-frequency bands, such as mu and beta. In this work, we demonstrated that the inclusion of cropped training significantly improves the classification of signals containing higher-frequency components, a result that aligns with prior findings in the literature.

It is important to note that when comparing raw data with the artifact-rejected dataset filtered in the 0.1–75 Hz range, we observe the same results: artifact rejection improves performance in four out of five networks, except EEGNet. However, when frequency filtering is applied to the raw data, the Shallow ConvNet without AR also outperforms the version where the FASTER method was applied. For the remaining networks, the performance relationship remains unchanged. Nevertheless, when cropped training was present, the overall picture changed. On one hand, the average classification accuracy of the networks decreases with this method. This decline can be attributed to the test set containing an extended range of data, making it more challenging for the classifiers to produce accurate results. On the other hand, via this method, we can acquire more data to train the classifier—as in the case of transfer learning. This extended version of the dataset is enough for the EEGNet and the Shallow ConvNet to learn from the higher frequency ranges—as expected from the literature. In investigating this aspect, it is shown that EEGNet and Shallow ConvNet are more capable of generalizing data, as they effectively leverage the variability introduced by cropped training. This demonstrates their ability to adapt to different temporal onsets in the EEG signals, a crucial factor in improving classification accuracy and model robustness.

Figure 11 demonstrates a notable consistency in the performance trends observed during cropped training across networks trained on frequency-filtered and FASTER-processed datasets, exhibiting a similar propensity for performance enhancement in comparison to the raw dataset. This consistency suggests that, in this case, the performance enhancement caused by the FASTER algorithm can be explained by the effect of frequency filtering. Moreover, if we compare the artifact rejection method to the frequency-filtered performance, the latter gets significantly higher accuracies in the vast majority of subjects. That means that with a simple frequency filter, we can achieve better performance during cropped training than with a complex AR method. Another important observation to note is that the subjects that had higher accuracies with the FASTER method compared to the raw data during cropped training differ from the subjects that were obtained by simple learning. This could be an effect of the phenomenon that during cropped training, different parts of the original signals are simultaneously presented, and neural networks can learn about other factors, some of which are filtered out during artifact rejection.

There are only a limited number of papers examining the subjects of the Physionet database regarding the effect of artifact rejection. Therefore, it is hard to give a thorough comparison. There are studies where filtering enhances the classification performance in various datasets [20,28]. Other articles report a decline in accuracy due to artifact rejection [21]. Subject dependence remains a significant factor, as evidenced not only in various articles exploring the BCI Competition IV Dataset 2a [25,48] but also in our study focusing on the Physionet database. Our findings highlight that the efficacy of the FASTER artifact rejection method in terms of classification accuracy is profoundly influenced by the specific subject under consideration.

For the case of frequency dependence, Hauke Dose et al. [42], who also examined the Shallow ConvNet’s accuracy on the Physionet database, concluded that this architecture tends to concentrate on the lowest part of the frequency domain. In their experiment, they analyzed the squared frequency responses of the learned temporal filters, and the mean focused on the lowest frequency range (below 10 Hz). These results correspond to our findings. The frequency results obtained through cropped training and the use of Shallow ConvNet and EEGNet align with the broader literature. As imagery movements are described to be mostly classifiable in the mu and beta ranges [49,50], it is expected that signals filtered between 5 and 75 Hz exhibit higher (or at least similar) accuracies compared to unfiltered signals.

The study reveals how small changes in the preprocessing pipeline can significantly impact classification accuracy, underscoring the need for tailored solutions in EEG-based BCI systems. As discussed by Xu et al. [51], while much progress has been made in neural interface research, translating these advancements into reliable, real-world applications remains challenging. This research contributes to bridging that gap by optimizing processing techniques that can enhance the practicality of BCIs in neurorehabilitation and beyond.

## 5. Conclusions

In conclusion, our research indicates that the FASTER method can enhance performance in a subject and network-specific manner. There are subjects where the application of AR comes with an efficiency increase, while in other cases, it comes with the deterioration of the results. Transfer learning proved to be effective in improving the performance of all networks in both raw and artifact-rejected data. However, it was noted that the accuracy of classification for artifact-rejected data did not improve as significantly as it did for the unfiltered data, resulting in less precision. Our findings also revealed an unexpected outcome from frequency filtering, as the tested networks demonstrated strong classification performance based on the low-frequency components during learning. Notably, we observed that higher frequency ranges were more discriminative in the case of EEGNet and Shallow ConvNet when cropped training was applied. In summary, the study underscores the intricate interplay between processing techniques and neural network performance, highlighting the necessity for tailored processing approaches designed for specific subjects and network architectures.

## Figures and Tables

**Table 1:** The accuracy of each classifier with and without artifact rejection, and the *p*-values of the significance of differences using Wilcoxon signed ranked test. The *p*-values determine the significance of differences between omitting or using the FASTER method for artifact rejection.

Classifier	Original Acc.	AR Acc.	*p*-Value
EEGNet	0.460	0.455	0.907
Shallow ConvNet	0.394	0.439	3.46 × 10^−17^
Conv2D Net	0.367	0.411	4.90 × 10^−21^
Conv3D Net	0.378	0.405	4.09 × 10^−09^
Multi-branch Conv3D Net	0.401	0.444	2.05 × 10^−19^

**Table 2:** The accuracy of each classifier on unfiltered data, with and without transfer learning, and the *p*-values of significance using the Wilcoxon test. The highest accuracies and the largest difference are highlighted in bold.

Classifier	Simple Acc.	TL Acc.	Difference	*p*-Value
EEGNet	**0.461**	0.587	0.126	1.50 × 10^−18^
Shallow ConvNet	0.394	**0.637**	**0.243**	5.83 × 10^−19^
Conv2D Net	0.366	0.528	0.162	7.78 × 10^−19^
Conv3D Net	0.378	0.56	0.182	7.14 × 10^−19^
Multi-branch Conv3D Net	0.401	0.561	0.16	1.30 × 10^−18^

**Table 3:** The accuracy of each classifier on artifact-rejected data, with and without transfer learning, and the *p*-value of significance using the Wilcoxon test. The highest accuracies and the largest difference are highlighted in bold.

Classifier	Simple Acc.	TL Acc.	Difference	*p*-Value
EEGNet	**0.455**	0.538	0.083	1.67 × 10^−17^
Shallow ConvNet	0.441	**0.559**	**0.118**	1.38 × 10^−18^
Conv2D Net	0.41	0.491	0.081	7.20 × 10^−17^
Conv3D Net	0.405	0.521	0.116	7.14 × 10^−19^
Multi-branch Conv3D Net	0.444	0.557	0.113	5.52 × 10^−18^

**Table 4:** Results of comparing the classification accuracies of neural networks when filters of certain frequency ranges are applied. The highest classification accuracy values for each frequency range are emphasized in bold.

Frequency Range	EEGNet	Shallow ConvNet	Conv2D Net	Conv3D Net	MB Conv3D Net
**0.1–5 Hz—Raw**	**0.482**	0.406	0.437	0.410	0.457
**5–75 Hz—Raw**	0.315	**0.362**	0.262	0.258	0.271
**0.1–5 Hz—AR**	0.452	0.405	0.421	0.436	**0.462**
**5–75 Hz—AR**	0.324	**0.381**	0.261	0.261	0.289

## Data Availability

We used the publicly available Physionet dataset, which can be accessed by the following link: https://physionet.org/content/eegmmidb/1.0.0/ (accessed on 13 December 2023).
